# The high-quality genome of pummelo provides insights into the tissue-specific regulation of citric acid and anthocyanin during domestication

**DOI:** 10.1093/hr/uhac175

**Published:** 2022-08-04

**Authors:** Zhihao Lu, Yue Huang, Sangyin Mao, Fangfang Wu, Yong Liu, Xiangqing Mao, Prakash Babu Adhikari, Yuantao Xu, Lun Wang, Hao Zuo, Muhammad Junaid Rao, Qiang Xu

**Affiliations:** Key Laboratory of Horticultural Plant Biology (Ministry of Education), College of Horticulture and Forestry Sciences, Huazhong Agricultural University, Wuhan, Hubei 430070, China; Key Laboratory of Horticultural Plant Biology (Ministry of Education), College of Horticulture and Forestry Sciences, Huazhong Agricultural University, Wuhan, Hubei 430070, China; Key Laboratory of Horticultural Plant Biology (Ministry of Education), College of Horticulture and Forestry Sciences, Huazhong Agricultural University, Wuhan, Hubei 430070, China; Science and Technology Innovation Research Center of Majia Pummelo, Guangfeng, Shangrao, Jiangxi 334000, China; College of Agronomy, Jiangxi Agricultural University, Nanchang, Jiangxi 330045, China; Service Center for Agriculture and Rural Area, Guangfeng, Shangrao, Jiangxi 334000, China; Key Laboratory of Horticultural Plant Biology (Ministry of Education), College of Horticulture and Forestry Sciences, Huazhong Agricultural University, Wuhan, Hubei 430070, China; Key Laboratory of Horticultural Plant Biology (Ministry of Education), College of Horticulture and Forestry Sciences, Huazhong Agricultural University, Wuhan, Hubei 430070, China; Key Laboratory of Horticultural Plant Biology (Ministry of Education), College of Horticulture and Forestry Sciences, Huazhong Agricultural University, Wuhan, Hubei 430070, China; Key Laboratory of Horticultural Plant Biology (Ministry of Education), College of Horticulture and Forestry Sciences, Huazhong Agricultural University, Wuhan, Hubei 430070, China; Key Laboratory of Horticultural Plant Biology (Ministry of Education), College of Horticulture and Forestry Sciences, Huazhong Agricultural University, Wuhan, Hubei 430070, China; State Key Laboratory for Conservation and Utilization of Subtropical Agro-Bioresources, College of Agriculture, Guangxi University, 100 Daxue Road, Nanning, Guangxi 530004, China; Key Laboratory of Horticultural Plant Biology (Ministry of Education), College of Horticulture and Forestry Sciences, Huazhong Agricultural University, Wuhan, Hubei 430070, China

## Abstract

Citric acid and anthocyanin contents were co-selected during *Citrus* domestication. Pummelo is a founding species in the *Citrus* genus, but the domestication of pummelo has not been well studied. Here, we compared the citric acid and anthocyanin contents of a low citric acid pummelo (*Citrus maxima* LCA) and its high citric acid variety (HCA) from the same cultivation area in China. Our study revealed that, unlike the LCA type, the HCA variety accumulated anthocyanin in the pericarp early in fruit development. To investigate the genetic basis of acid and anthocyanin enrichment in HCA pulp and pericarp, respectively, we generated a chromosome-scale HCA genome using long-read sequence reads and Hi-C sequencing data. Transcriptome analysis and transient overexpression assays showed that the accumulation of citric acid and anthocyanin was associated with high expression of *CgANTHOCYANIN1* (*CgAN1*), and two different *MYBs* transcription factors (*CgPH4* and *CgRuby1*), respectively. Moreover, the *CgAN1* promoter was more methylated in the LCA pulp than in the HCA pulp. Treatment with a DNA methylation inhibitor, 5-azacytidine, alleviated the *CgAN1* promoter hypermethylation in the LCA pulp, leading to increased *CgAN1* expression and citric acid content. This study provides a new high-quality pummelo genome and insight into the molecular mechanism behind the change in tissue-specific citric acid and anthocyanin accumulation during pummelo domestication and provides a conceptual basis for precise genetic manipulation in fruit flavor breeding.

## Introduction

Citric acid and anthocyanin contribute to the flavor and color of *Citrus* fruits, respectively. Differentiated patterns of fruit color and acidity were formed during *Citrus* domestication. The citric acid content is generally higher in wild *Citrus* accessions than in cultivars. Likewise, wild fruit pericarps and flowers have higher anthocyanin contents than those of modern cultivars [1, 2].

Acidity and anthocyanin accumulation are co-regulated in fruit crops, including citrus, apple (*Malus domestica*), and ornamental crops, including petunia (*Petunia* × *atkinsiana*) [1–3]. Among these species, MYB-bHLH-WD40 (MBW) complexes, including *PH4* (encoding an MYB transcription factor), *AN1* [encoding a basic helix–loop–helix (bHLH)], and *AN2* (encoding an MYB transcription factor), synergistically regulate acidity and anthocyanin accumulation [1, 4, 5]. In petunia, the AN1-AN2 complex positively regulates anthocyanin accumulation, and the AN1-PH4 complex positively regulates vacuole acidification and thus affects pH [6]. In apple, *MdbHLH3*, a homolog of the *AN1* gene, also interacts with *MdMYB1*, a homolog of the *AN2* gene, to promote anthocyanin and malate accumulation in fruit flesh [2]. In citrus, the homolog genes of the AN1-PH4 and AN1-Ruby1 (*Ruby1* is a homolog of *AN2*) complexes are associated with acidity and anthocyanin accumulation [7, 8]. Furthermore, loss of function of *AN1* is associated with low acidity and loss of anthocyanin in citrus [3].

In contrast to the better understanding of the transcriptional regulation of citric acid and anthocyanin accumulation, the effects of epigenetic modification on these traits are less known. In apple, hypermethylation in the promoter of *MdMYB10*, which encodes an anthocyanin activator, inhibits its transcription [9–11]. However, the mechanism of tissue-specific citric acid and anthocyanin accumulation is unclear.

Majia pummelo (*Citrus maxima*, ‘Majiayou’) is a landrace commercially cultivated in Guangfeng County, Jiangxi province, China, since the Ming Dynasty (1472 AD) according to the Guangfeng County records. Majia pummelo was named a China National Geographical Indications Product for its succulent fruit and its contribution to the local agricultural industry. Thecommercial cultivar of Majia pummelo has low citric acid content and does not accumulate anthocyanin in the pericarp [it is hereafter referred to as the low citric acid (LCA) cultivar]. In addition, LCA is also an edible fruit for type II diabetes [12]. A new variety of Majia pummelo was identified recently in the same region; it accumulates higher contents of citric acid in the pulp [it is hereafter referred to as the high citric acid (HCA) variety] and accumulates anthocyanin in the fruit pericarp at the early developmental stage. HCA shows a phenomenon of tissue-specific accumulation of citric acid and anthocyanins in fruits.

Here, we aimed to understand the mechanism by which high citric acid and anthocyanin accumulate in the HCA variety. A high-quality chromosome-scale genome was assembled *de novo* and transcriptome analyses were performed on the pericarp and pulp of the two varieties of Majia pummelos. The candidate genes involved in tissue-specific accumulation of citric acid and anthocyanins were identified. Through expression analysis and transient overexpression assays, we conclude that the high expression level of the core MBW complex members *CgAN1* and *CgRuby1* leads to anthocyanin accumulation in HCA pericarp, and the high expression level of *CgAN1* and *CgPH4* in HCA pulp leads to enrichment in citric acid. Notably, we validated that *CgAN1* promoter demethylation leads to increased self-expression and citric acid contents.

## Results

### Identification of a low citric acid pummelo and its high citric acid variety

**Figure 1:**
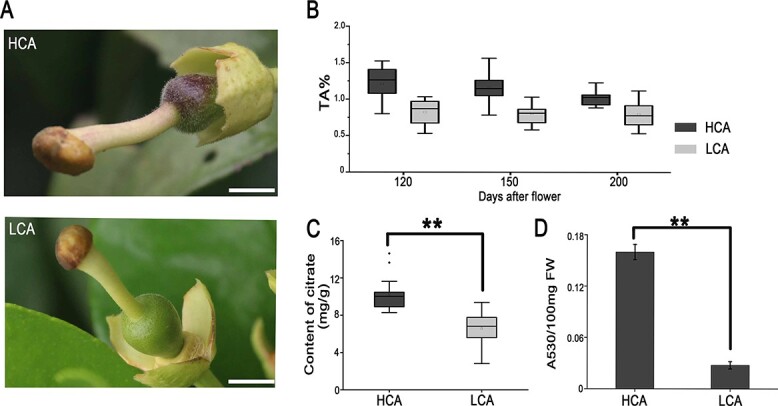
Tissue-specific citric acid and anthocyanin accumulation in the HCA and LCA varieties. (A) Phenotypes of HCA and LCA pericarps at the early developmental stage. The stamens were manually removed before taking photographs. (B) Titratable acid (TA) content in HCA and LCA pulp at 120, 150, and 200 DAF. Data are mean ± standard error (*n* = 15–23 independent samples from different trees). (C) Citric acid content in HCA and LCA pulp at 120 DAF (*n* = 15 independent samples from different trees). (D) Anthocyanin content in HCA and LCA pericarp at the early developmental stage. Data are mean ± standard error (*n* = 3 biologically independent replicates). Scale bars = 1 cm. Asterisks indicate significant differences: ^**^P < .01.

Pummelo (*C. maxima*) is one of the three progenitor species in the *Citrus* genus. LCA, a type of low citric acid Majia pummelo, has been cultivated for >500 years in Guangfeng county, Jiangxi province, China. It is worth noting that a high citric acid Majia pummelo (HCA) appeared in this period during the cultivation process. The most obvious differences between the HCA and the LCA pummelos are in the citric acid content in the pulp and anthocyanin content in the pericarp (Fig. 1A). The levels of citric acid and titratable acid were significantly higher in HCA pulp compared with LCA pulp. The difference in titratable acid was highest at 120 days after flowering (DAF) (Fig. 1B and C). The HCA fruit pericarp began to accumulate anthocyanin at 10 DAF, and the anthocyanin content in HCA and LCA types also showed a significant difference at this stage (Fig. 1A and D, Supplementary Data Fig. S1).

### *De novo* assembly of the HCA pummelo genome and comparison with other citrus genomes

**Figure 2:**
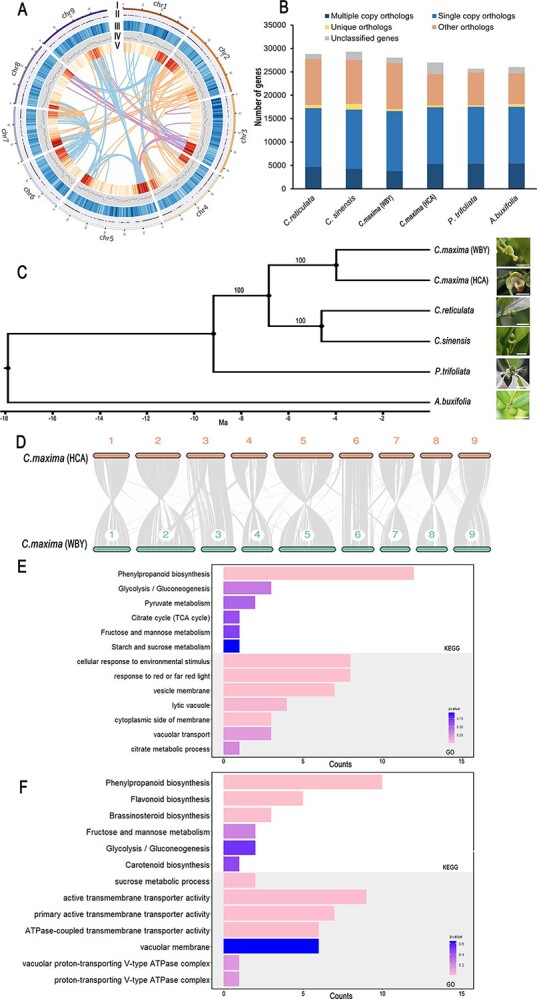
Genome comparison among the six *Citrus* species. (A) HCA pummelo genome characterization. (I) Chromosome ideogram. (II) Unique genes of HCA. (III) Distribution of TEs (window size 500 kb). (IV) GC content between 30 and 40% (window size 500 kb). (V) Gene density (window size 500 kb). (B) Classification of homologous gene families in the six *Citrus* species identified through OrthoFinder. (C) HCA phylogenetic status among the six *Citrus* species identified using an ML phylogenetic tree based on single-copy orthologous genes. (D) Syntenic blocks between HCA and WBY. (E) Main biological functions identified as being involved in the PAVs located in the gene body regions. (F) Main biological functions identified as being involved in the PAVs located in the promoter regions.

To investigate the tissue-specific accumulation of citric acid and anthocyanin in fruit, a high-quality genome of the HCA variety was generated by Nanopore long-read sequencing combined with Illumina short-read sequencing and high-throughput chromosome conformation capture (Hi-C) sequencing (Table 1). To verify the assembly quality, we confirmed that 99.55% of HCA Illumina sequences could be mapped to the assembled HCA genome. The error rate was <0.01% based on the heterozygous single-nucleotide polymorphism (SNP) rate calculation. Assembly completeness was 98.1%, as assessed by Benchmarking Universal Single-Copy Orthologs (BUSCO v3.0.2) based on 1348 conserved plant genes. In terms of gene model predictions, the *ab initio* gene predictions, homology searches, and RNA-seq analysis were integrated. In total, 26 988 genes were identified in HCA, and the protein coding genes were annotated functionally by Gene Ontogeny (GO) terms, motifs, domains, and their related pathways. An overview of the gene density, GC content, unique genes, detected syntenic blocks, and transposable element (TE) density, are presented in Fig. 2A.

**Table 1:** Comparison of HCA and WBY genome assembly.

	HCA (diploid pummelo)	WBY (haploid pummelo) [13]
Assembled size (bp)	367 609 127	345 744 738
Contig N50 (bp)	3 801 589	2 182 545
Contig N90 (bp)	514 586	70 069
Longest contig (bp)	15 127 647	10 624 441
Scaffold N50 (bp)	38 734 001	4 210 623
Scaffold N90 (bp)	28 405 889	565 389
Longest scaffold (bp)	56 996 108	14 289 975
Anchored length (bp)	345 131 750	301 956 574
TE ratio	57.07%	45.84%

We compared the genomic characteristics of the HCA variety with those of several other *Citrus* species: sweet orange (*Citrus sinensis*), pummelo cultivar ‘Wanbai pummelo’ (WBY), Mandarin orange (*Citrus reticulata*), *Atalantia buxifolia*, and trifoliate orange (*Poncirus trifoliata*). The many collinear blocks indicated consistent gene collinearity between the HCA and WBY genomes. [13] (Fig. 2D). Then, we compared putative orthologs and paralogs among the *Citrus* species and identified 24 618 gene families based on gene family clustering. Of these, 14 257 gene families were shared by the six *Citrus* species, and 9341 of these families were single-copy (Fig. 2B). These single-copy ortholog genes were aligned and conservative sequences were extracted to generate a maximum likelihood (ML) phylogenetic tree. HCA and WBY were clustered in the same subgroup (Fig. 2C). The number and type of TEs showed significant differences among six genomes (Supplementary Data Fig. S2A). We identified 2116 presence/absence variants (PAVs) between WBY and the HCA Majia pummelo. Among them, 447 PAVs were located in promoter regions and 428 PAVs were located in gene body regions. KEGG (Kyoto Encyclopedia of Genes and Genomes) pathway and GO enrichment analysis indicated that these variations, both located in the promoter or the gene body, were correlated with fundamental biological processes, such as the phenylpropane pathway and carbohydrate and citrate metabolism. Furthermore, there were significant variations in genes located in the membranes of vacuoles and vesicles, these being mostly related to acid transport and storage (Fig. 2E and F). This suggests that there are more variations in the flavor-related pathways in the HCA genome compared with WBY.

### Investigation of genetic relationships between the two types of Majia pummelos

**Figure 3:**
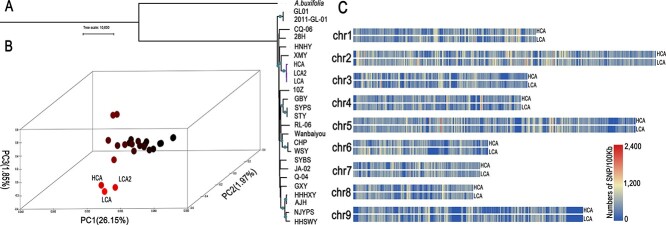
Genetic relationship among high-acid and low-acid pummelos and their associated candidate genes. (A) Phylogenetic tree of the 25 pummelo accessions. (B) Resequencing-wide PCA of the 25 accessions from Southeast, Southwest, and Central China. (C) Comparison of heterozygosity between HCA and LCA pummelos.

The pulp shape and development environment of the HCA and LCA pummelos are extremely similar (Supplementary Data Fig. S3). To understand the genetic background between HCA and LCA, the neighbor-joining (NJ) phylogenetic tree based on 4dTV, principal component analysis (PCA), identity by state (IBS), and the value of genetic relationship calculated by GCTA (genome-wide complex trait analysis) were generated. Phylogenetic analysis and PCA both distinguished HCA and LCA from other pummelos and grouped them into a single group (Fig. 3A and B). The IBS value was 0.86, and the GCTA value of the genetic relationship was 0.5 between HCA and LCA, further indicating that their genetic backgrounds are quite similar (Supplementary Data Figs S4A and B). A comparison of genome heterozygosity of the HCA and LCA pummelos showed the existence of more than one dissimilar region per chromosome, according to genome-wide SNP information. These results indicate that the relationship between HCA and LCA is not a simple bud mutation (Fig. 3B). In addition, to screen out HCA in the early stage of growth to better meet production needs, two InDel markers were designed (Supplementary Data Fig. S2B and C).

### High expression of *CgAN1*, *CgRuby1*, and *CgPH4* contribute to citric acid and anthocyanin tissue-specific accumulation

**Figure 4:**
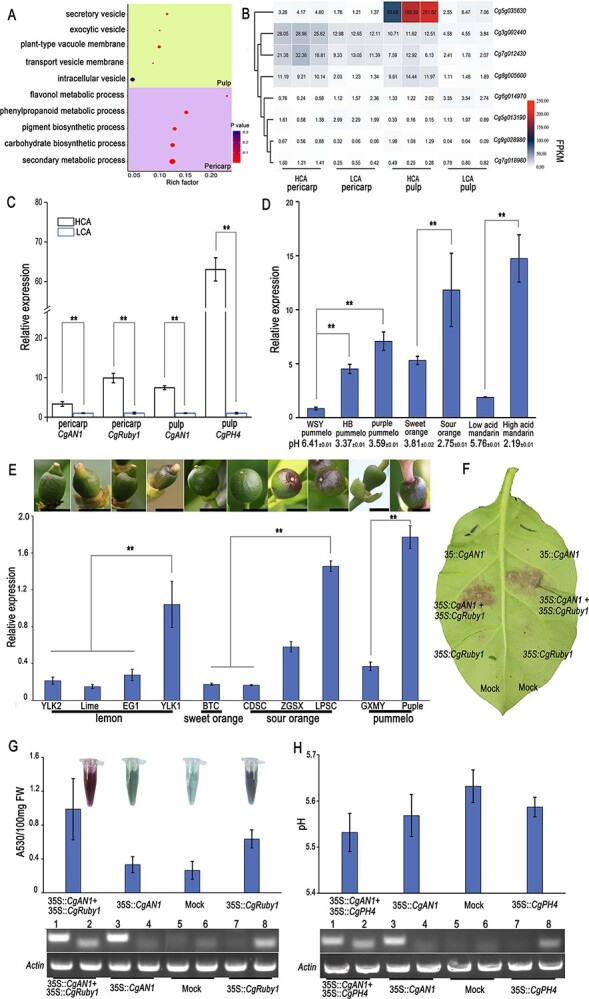
Identification of citric acid- and anthocyanin-related genes. (A) GO enrichment of DEGs in pulp and pericarp. Circle size indicates the number of enriched genes. (B) Overlapping differentially expressed transcription factors in HCA and LCA pericarp and pulp. (C) *CgAN1*, *CgPH4*, and *CgRuby1* expression analysis in pulp and pericarp. (D) *CgAN1* expression analysis in high-acid citrus pulp and low-acid citrus pulp. High-acidity pulp and low-acidity pulp are indicated by pH, shown below the *x* axis. (E) *CgAN1* expression analysis in purple citrus pericarp and normal citrus pericarp. Scale bars = 1 cm. Data are mean ± standard error (*n* = 3 biologically independent replicates). ^**^P < .01. (F) Transient functional assay of *CgAN1* and *CgRuby1*. (G) Analysis of anthocyanin content in transient tobacco leaves. The first row of the electrophoresis gel shows a semiquantitative real-time polymerase chain reaction (RT–PCR). Lanes 1, 3, 5, and 7 indicate the expression level of *CgAN1*. Lanes 2, 4, 6, and 8 indicate the expression level of *CgRuby1*. (H) Analysis of pH in transient tobacco leaves. The first row of the electrophoresis gel shows RT–PCR. Lanes 1, 3, 5, and 7 indicate the expression level of *CgAN1*. Lanes 2, 4, 6, and 8 indicate the expression level of *CgPH4*. Data are mean ± standard error (*n* = 8 biologically independent replicates).

To explore the molecular basis of the higher citric acid and anthocyanin contents in HCA pulp and pericarp, respectively, we profiled gene expression in the pulp at 120 DAF and pericarp at 10 DAF. Among the 1416 differentially expressed genes (DEGs) found in pulp, 640 were upregulated and 776 were downregulated (Supplementary Data Table S1). Numerous genes associated with vesicles and vacuoles were impacted, according to a GO analysis (Fig. 4A). Additionally, the expression of citric acid biosynthesis and transport structural genes was significantly higher compared in HCA pulp than in LCA pulp (Supplementary Data Fig. S5A, Supplementary Data Table S2). Changes in the expression of upstream transcription factors may cause changes in the expression of many citric acid pathway structural genes. Notably, the MYB gene *CgPH4* (Cg2g000510) and the bHLH gene *CgAN1* (Cg5g035630), highly correlated with promotion of high acid [18], are highly related to acid accumulation and showed higher expression in HCA pulp compared with LCA pulp (Fig. 4C).

In the pericarp, we discovered 1210 DEGs, comprising 729 upregulated and 481 downregulated genes (Supplementary Data Table S3). The most affected were pigment biosynthesis and phenylpropanoid metabolism, according to GO analysis (Fig. 4A). Furthermore, expression profiling of these pericarp genes illustrated that anthocyanin pathway structural genes were considerably elevated in HCA pericarp (Supplementary Data Fig. S5B, Supplementary Data Table S4). These findings also suggested that upstream transcription factors may result in these gene expression changes. Notably, *CgRuby1* (Cg6g018450), an MYB transcription factor that promotes anthocyanin biosynthesis [5, 14, 15], and a bHLH transcription factor gene, *CgAN1*, which is highly correlated with anthocyanin accumulation [4], were both highly expressed in the HCA pericarp compared with the LCA pericarp (Fig. 4C).

We found 232 DEGs overlapped between the pericarp and pulp transcriptomes, among which 8 were putative transcription factors as predicted by the Plant Transcription Factor Database (http://planttfdb.gao-lab.org) (Fig. 4B, Supplementary Data Table S5). To determine whether these eight candidate TFs are related to citric acid and anthocyanin accumulation, we compared the expression profiles of four other pummelo cultivars (HB, WSY, PP, and NP) at the same developmental stage (from the Citrus Pan-genome to Breeding Database: http://citrus.hzau.edu.cn/). The transcriptome results showed that *CgAN1* expression was positively correlated with anthocyanin and citric acid accumulation, while the expression of the remaining seven transcription factors did not show a correlation with the anthocyanin and citric acid contents (Supplementary Data Fig. S6C). The findings are consistent with previous reports that *PH4* and *Ruby1* promote citric acid and anthocyanin accumulation, respectively, and that *AN1* is associated with the accumulation of both [1, 3, 5]. We further investigated the expression patterns of *CgPH4*, *CgAN1*, and *CgRuby1* in HCA and LCA pericarp and pulp using RT–qPCR and observed that their expression was significantly higher in HCA, which could be the reason behind the higher citric acid and anthocyanin accumulation in this variety (Fig. 4C).

To support the contribution of high expression of *CgAN1* to citric acid accumulation and anthocyanin production, we determined *CgAN1* expression in pulp from high- and low-acid citrus species, as well as young-stage pericarp from purple pericarp and non-purple pericarp citrus species (Fig. 4D and E). The results confirmed that *CgAN1* expression was considerably higher in purple pericarp and high-acid pulp than in control. To confirm the contribution of *CgAN1*, we performed transient expression assays in tobacco (*Nicotiana tabacum*) leaves. The contents of anthocyanin in the *CgAN1* and *CgRuby1* co-infiltration tobacco leaves were higher than those in single-infiltration *CgAN1* or *CgRuby1* tobacco leaves. (Fig. 4F and G). The pH value in the *CgAN1* and *CgPH4* co-infiltration tobacco leaves was lower than that in single-infiltration *CgAN1* or *CgPH4* (Fig. 4H)*.* These results indicated that *CgAN1* can promote acidity and anthocyanin accumulation*.*

### Hypermethylation in the *CgAN1* promoter is correlated with decreased citric acid content in fruit pulp

To further explore the mechanism of anthocyanin and acid differential accumulation, we isolated the promoter and coding sequences of *CgAN1*, *CgPH4*, and *CgRuby1* in HCA and LCA pummelos. There were no differences in the coding sequences of *CgAN1*, *CgPH4*, and *CgRuby1* between HCA and LCA pummelos. The high similarity of the AN1, PH4, and Ruby1 protein sequences among HCA, lemon, apple, and petunia, especially the MYC domain, which interacts with MYB, and the [DE]Lx2[RK]x3Lx6Lx3R domain, which interacts with bHLH, indicated that CgAN1, CgPH4, and CgRuby1 were correlated with acid and anthocyanin accumulation together in HCA pulp and pericarp (Supplementary Data Figs S7 and S8). However, the *CgPH4* promoter sequences differed in HCA and LCA. The promoter regions of *CgAN1* and *CgRuby1*, on the other hand, were the same (Supplementary Data Fig. S9). Furthermore, the promoter sequence differed between the two *CgPH4* alleles in the HCA pummelos, whereas LCA was found to be homozygous for one of the HCA alleles. The activity of the unique *CgPH4* promoter in HCA pummelos was slightly higher than that of the common *CgPH4* promoter. (Supplementary Data Fig. S10).

**Figure 5:**
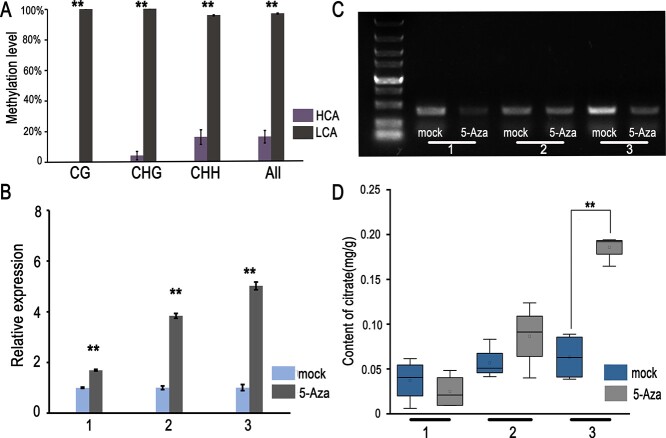
Methylation level in the *CgAN1* promoter affects citric acid content in fruit pulp. (A) Cytosine methylation levels in the −1 to −399 bp region of the *CgAN1* promoter in the pulp. The *y*-axis shows the percentage of methylation of each cytosine in the clones; five to seven clones were measured. (B) Level of *CgAN1* expression in LCA pulp after treatment with water and 5-Aza. (C) *CgAN1* promoter methylation level in LCA was analyzed using the methylation-sensitive restriction endonuclease HaeIII after treatment with 5-Aza. (D) Citric acid content in LCA pummelo pulp after treatment with 5-Aza. 1, 2, and 3 are three independent biological duplications of 50 mM 5-Aza and water treatment. Data are mean ± standard error (*n* = 3 biologically independent replicates). ^**^P < .01.

The methylation levels of the *CgAN1*, *CgPH4*, and *CgRuby1* promoters were then investigated. The methylation levels of the *CgPH4* promoter in pulp and the *CgAN1* and *CgRuby1* promoters in pericarp were similar (Supplementary Data Fig. S11). However, the methylation level of the −1 to −399 bp region in the *CgAN1* promoter in HCA pulp was considerably lower than that in LCA pulp (Fig. 5A). To investigate whether the *AN1* promoter methylation level impacts its transcription in the pulp, we injected 50 mM 5-azacytidine (5-Aza), a DNA methylation inhibitor, and water separately into the pulp on each side of LCA fruits (Supplementary Data Fig. S11D), four times in total, 2 days apart each time, using 12 pummelos as three biological duplications. We then collected tissue samples and found that *CgAN1* expression increased significantly in 5-Aza-treated samples compared with the water-treated samples (Fig. 5B). Next, we digested the DNA with HaeIII, a methylation-sensitive restriction endonuclease, followed by PCR. The results verified that 5-Aza decreased the methylation level of the −1 to −399 bp region in the *CgAN1* promoter (Fig. 5C, Supplementary Data Fig. S11E). Furthermore, the citric acid content tended to increase in the pulp of the 5-Aza-treated samples compared with that in the water-treated control (Fig. 5D). These results indicated that the high expression levels of *CgAN1* and *CgRuby1* in the HCA pericarp are responsible for anthocyanin accumulation, and the high expression levels of *CgAN1* and *CgPH4* in the HCA pulp are responsible for citric acid accumulation. Also, the high level of *CgAN1* promoter methylation in LCA pulp resulted in low acid content.

## Discussion

**Figure 6:**
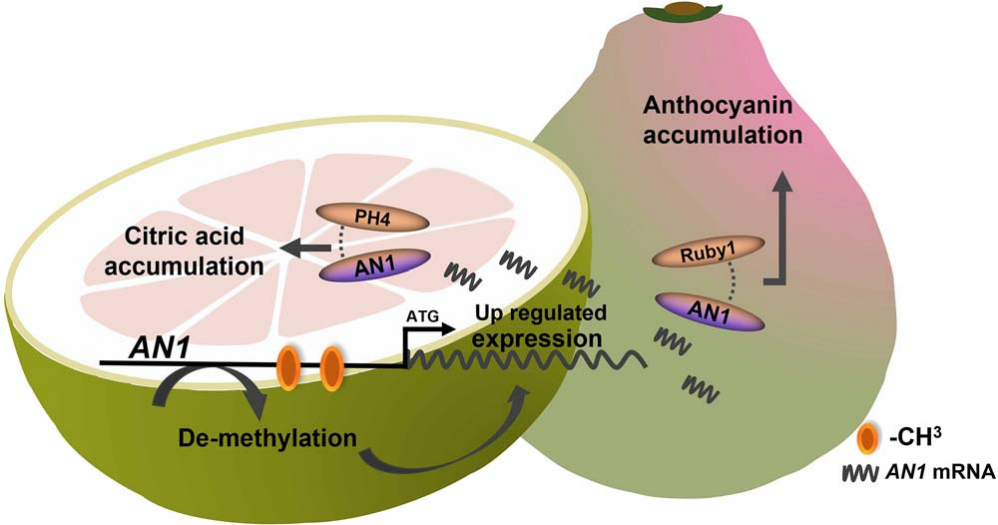
Model of the regulatory mechanism of tissue-specific anthocyanin and citric acid accumulation. In the HCA fruit pulp, de-methylation of the *CgAN1* promoter results in increased gene expression and ultimately increased accumulation of citric acid. In the HCA pericarp, high expression of *CgAN1* and *CgRuby1* leads to high anthocyanin accumulation.

*Citrus* quality traits such as acid, color, and bitterness were under selection during domestication, and acid and anthocyanin are usually co-selected [1, 16]. Acidity affects the taste of fruit. Sour pummelo, usually used as a rootstock, is a wild germplasm that occurs widely in South China. There are also abundant landraces of pummelo with varying degrees of fruit acidity [17]. The HCA pummelo used in this study is a variety of the LCA Majia pummelo cultivar. Aside from their citric acid and anthocyanin contents, HCA and LCA are very similar in evolutionary status and genetic relationship. However, HCA accumulates anthocyanin in the fruit pericarp during the early development stage, in contrast to our earlier work on a purple pummelo, which accumulates anthocyanins in later fruit developmental stages [5]. This could be the response of *CgRuby1* and downstream anthocyanin structural genes to diverse developmental signals. Future studies need to elucidate why anthocyanins accumulate in different developmental stages. In this study, however, we showed that hypermethylation of the *CgAN1* promoter is associated with decreased citric acid content in fruit pulp, and the CgAN1-CgRuby1 complex promotes anthocyanin accumulation in pericarp (Fig. 6).

The current study has revealed that the phenomenon of tissue-specific accumulation of citric acid and anthocyanin could be explained by the tissue-specific expression of the MBW complex. *Ruby1* is a key regulator of anthocyanin accumulation in *Citrus* [5, 18]. Many citrus species have lost anthocyanin due to truncation of Ruby1 or alterations in the *cis*-elements of the Ruby1 promoter [19]. We observed that *CgRuby1* expression was higher in the HCA pummelo pericarp than in the LCA pummelo pericarp. However, the *CgRuby1* coding sequence was the same in the HCA, LCA, and purple pummelo (Supplementary Data Fig. S8) [5]. The KPXPR(S/T)F motif, also named the S6A motif, on the C terminus of Ruby1 is highly conserved with homologs in other fruit crops, including apple MYB10 and petunia AN2 (Supplementary Data Fig. S8) [20]. Importantly, a transient overexpression assay revealed that *CgRuby1* of HCA may also increase anthocyanin accumulation. In addition, no significant differences in the sequence and methylation level of the *CgRuby1* promoter in the high- and low-acid pummelo pericarps were observed, implying that *CgRuby1* expression might be transcriptionally regulated by additional transcription factors at the early fruit developmental stage. In the pulp, *CgPH4* expression was found to be significantly higher in high-acid pummelo than in low-acid pummelo, and the *CgPH4* coding sequence is also highly conserved among citrus, petunia, and some other fruit trees (Supplementary Data Fig. S7C). *PH4* was first identified in petunia and is highly correlated with acidification in citrus [21]. Furthermore, a transient overexpression assay revealed that *CgPH4* of HCA can enhance acidity. These results indicate that the higher level of *CgPH4* expression is likely involved in citric acid accumulation in high-acid pummelo.

*AN1* promotes citric acid and anthocyanin accumulation, and exhibits a typical example of pleiotropy. *AN1* was originally shown to improve anthocyanin and acid accumulation in petunia. Itdirectly activates the *Dihydroflavonol 4-Reductase* (*DFR*) promoter to boost anthocyanin production. Furthermore, the AN1-MYB complex has a stronger promoting effect on both anthocyanin biosynthesis and vacuolar acidity than MYB alone [4, 21]. In citrus, *AN1* (also named Noemi) is associated with citric acid accumulation in pulp and with pigmentation (anthocyanin and proanthocyanin) in leaves, seeds, and flowers [7]. The loss of CgAN1 function leads to the loss of anthocyanin and citric acid [3, 22]. Here, we detected high abundance of *AN1* in high-acid pummelo pulp and high-anthocyanin pummelo pericarp. The coding sequence of *CgAN1* from Majia pummelo is highly similar to that in Faris lemon (*Citrus limon*), petunia, and apple. Additionally, its MYC domain, which interacts with MYB, is also largely conserved among these species (Supplementary Data Fig. S7A). In addition, transient overexpression assays indicated that co-infiltration with *CgAN1*-*CgPH4* and *CgAN1*-*CgRuby1* results in lower pH and higher anthocyanin contents than single infiltration, indicating that *CgAN1* is a shared key regulator for anthocyanin accumulation in the pericarp and citric acid accumulation in the pulp of pummelo.

Epigenetic modifications have played crucial roles during the domestication of crops such as blood orange, apple, tomato (*Solanum lycopersicum*) and cotton (*Gossypium hirsutum*) [23–25]. In blood orange, hypomethylation of the *Ruby1* promoter and the *DFR* promoter is associated with their increased expression under cold stress, and *DEMETER-LIKE1* (*DML1*) might have a vital role [26]. Hypermethylation of the *PcMYB10* promoter leads to the formation of green-skinned spots in red-skinned pear (*Pyrus communis*). This was confirmed by creating site-specific hypermethylation in the *PcMYB10* promoter through virus-induced gene silencing [27]. In apple, Argonaute4 (AGO4) directly binds to the *MdMYB1* promoter to mediate methylation modifications [9]. Aside from the effect of epigenetic modifications on pigmentation, the high methylation levels in the *colorless nonripening* (*Cnr*) promoter, a critical gene involved in fruit ripening, diminishes its expression and inhibits fruit maturation [28]. Furthermore, methylation was involved in the photoperiod adaptation of allotetraploid cotton to expand its cultivation range [23]. Here, we determined that the −1 to −399 bp region in the *CgAN1* promoter was significantly less methylated in the high citric acid pummelo pulp than in the low citric acid pummelo pulp. Furthermore, treatment with the DNA methylation inhibitor 5-Aza significantly increased *CgAN1* expression led by the decreased methylation level of the −1 to −399 bp region in the *CgAN1* promoter. In parallel, the 5-Aza treatment also increased the citric acid content in the LCA pulp. As a result, we demonstrated that the methylation level of the *AN1* promoter plays a vital role in regulating citric acid content. Our findings also provide a theoretical basis for understanding the impact of epigenetic modifications in pummelo during domestication.

Taken together, our high-quality genome of pummelo provides a new resource for gene identification and genetic improvements in citrus. Transient overexpression assays indicated that the high expression levels of *CgAN1* and *CgRuby1* in the pericarp and high expression levels of *CgAN1* and *CgPH4* in the pulp contributed to the tissue-specific accumulation of anthocyanin and acidity, respectively. Lastly, the methylation level of the *CgAN1* promoter regulates its expression, with decreasing methylation resulting in increased expression and, in turn, leading to higher citric acid accumulation. Discovering the mechanism of de-methylation in the *CgAN1* promoter and the upstream regulatory factors of the MBW complex will help us understand the reasons for the change in acid and anthocyanin contents during the domestication process and will aid in the breeding of high-quality horticultural crops in the future.

## Materials and methods

### Plant materials

The HCA and LCA pericarps as well as respective pulps were collected from Guangfeng County, Jiangxi province, China. Pummelo pulp was collected at 120, 150, 200, and 210 DAF. Fruit pericarp was collected at 10, 120, 150, 200, and 210 DAF. Three fruit trees were used as a biological duplicate. These materials were collected and frozen using liquid nitrogen.

### Citric acid, pH value, and anthocyanin extraction and quantification

Anthocyanin extraction and quantification were carried out by following a previously published method [29] with minor modifications. The powder of pericarp was mixed with a buffer containing methanol with 1% HCl (v/v). After the powder had been entirely decolorized, it was centrifuged at 12 000 rpm for 15 minutes at 4°C. Anthocyanin was quantified at 530 and 657 nm using a spectrophotometer and converted into its fresh weight content using the following formula: anthocyanin content = absorbance at 530 nm – 0.25 × absorbance at 657 nm/fresh weight.

Two techniques were used to determine organic acids. The content of citric acid in Fig. 1C was determined by gas chromatography as described before [30], whereas the contents of citric acid in Fig. 5D and Supplementary Data Fig. S1A were evaluated by gas chromatography–mass spectroscopy as described previously [31]. In brief, 200 mg pericarp and pulp powder was homogenized in 2700 μl methanol followed by the addition of 300 μl ribitol (0.2 mg/ml) solution. After derivatization, the samples were examined following Tan *et al*. [31]. The pH value of pulp powder was measured using a pH meter after homogenizing 0.3-g samples in 3 ml water.

### Genome assembly and genome annotation

HCA genome features were estimated based on Illumina reads using GCE (v1.0.2) software. Approximately 59.7 Gb of Nanopore raw data was used to assemble the genome. NECAT (v20200803) was firstly used to assemble the genome and retrieve raw contigs. Raw contigs were iteratively corrected three times by Racon (v1.4.7). Finally, Illumina reads were used to further polish the assembly by Nextpolish (v1.3.1). To assemble contigs to chromosome-level scaffolds, Hi-C reads were firstly mapped to the contigs using Juicer (version 1.6). Contigs were then anchored, oriented, and sorted using 3d-dna (v190716).

For TE annotation, RepeatModeler (v2.0.1) was first used to build a repeat library. The repeat library was then combined with the Repbase plant database. Finally, RepeatMasker (v4.0.9) was used to mask the repeat sequence of the genomes. Gene model annotations were performed by integrating *ab initio* gene prediction, homology research, and RNA analysis. For *ab initio* gene exploration, Augustus (v3.3.2) and GlimmerHMM (v3.0.4) were used to predict genes. Then, published citrus proteins were used to confirm gene structures by GenomeThreader (v5.4.0). Finally, RNA-seq was used to align to the genomes by Hisat2 (v2.1.0) and the transcripts were assembled by Trinity (v2.8.5). The assembled transcripts were used by PASA (v2.3.3) to predict transcripts. All these results were combined by EVM (v1.1.1) to construct the main structure of protein genes, and PASA was used to predict annotation of the UTR and alternative splicing isoform types.

### Phylogenetic and genetic relationship analysis

The phylogenetic tree of *Citrus* genomes was built using single-copy orthologous genes retrieved via OrthoFinder (v2.5.4) [32]. Tandem single-copy orthologous genes were aligned by Muscle (v3.8.1551) and Gblocks (v0.91b) to extract conserved regions with default settings. The ML phylogenetic tree was generated by RAxML (v8.2.12) with 1000 bootstraps [33]. The NJ phylogenetic tree of pummelos was built based on 4dTV. OrthoFinder with default parameters was also used to identify gene collinear blocks between HCA and WBY.

BWA (v0.7.15) and GATK (v4.1.2.0) were used for SNP calling [34]. SnpEff (v5.0e) was used to extract 4dTV from an annotated vcf file [35]. The NJ phylogenetic tree was built by MEGAx with 1000 bootstraps [36]. IBS was calculated by plink (v1.90b6.10) and the value of individuals genetic relationship based on SNP was calculated by GCTA (v1.93.3) with default settings respectively [37]. The accession numbers are listed in Supplementary Data Table S6.

### Transcriptome analysis and transient overexpression assay

The RNA of HCA and LCA fruit pericarp and pulp were extracted using a TRIzol RNA extraction kit (Takara). RNA-seq analyses were performed on these samples. TopHat (v2.1.0) and Cufflinks (v2.2.1) were used to calculate FPKM (fragments per kilobase of transcript per million mapped reads) [38, 39]. Fold change >2 or fold change <0.5 and *P* <.05 were set as the threshold for screening DEGs. The DEG expression patterns were shown using heat maps of TBtools (v1_098761) [40]. edna clusterProfiler (v4.4.4) was used for GO and KEGG enrichment [41]. We used an LC480 instrument (Roche) for RT–qPCR. Relative expression levels were calculated by the 2^−ΔΔCt^ method. Analysis of differences between sequences was performed through promoter and coding sequence amplification using genomic DNA and cDNA as templates, respectively. Sequence amplification was performed using DNA polymerase and then ligated the fragments to p-TOPO vector for subsequent experiments. The primer of two markers used to identify HCA and LCA are provided in Supplementary Data Table S7. To generate *CgRuby1*, *CgPH4*, and *CgAN1* overexpression vectors, these genes' coding sequences from HCA were amplified and recombined to pK7WG2D vector using the one step LR Clonase (enzyme mix) reaction. Constructed vectors were transformed into *Agrobacterium tumefaciens* strain GV3101 and a transient overexpression assay was carried out according to previous reports [8].

### β-Glucuronidase assays

The two types of promoters of *CgPH4* were cloned into DX218G through homologous recombination, to prepare the *CgPH4* promoter-driven β-glucuronidase (GUS) expression cassette. Vectors were constructed and converted into *A. tumefaciens* strain GV3101-pSoup-p19. The *A. tumefaciens* strain GV3101 harboring HCA_*PH4*pro-1:*GUS* and LCA_*PH4*pro:*GUS* were grown overnight to OD_600_ = 0.6–0.8. Finally, *A. tumefaciens* strain GV3101 was resuspended in liquid Murashige and Skoog (MS) medium containing 2% sucrose, pH 5.7–5.8. The tobacco leaves were injected with an *Agrobacterium* suspension. Histochemical staining and fluorometric assays were performed after 3 days [5, 42]. Tobacco leaves were submerged in X-gluc (5-bromo-4-chloro-3-indolyl-β-d-glucuronide) overnight at 37°C, followed by decolorization. Protein was extracted as described previously [43]. Protein concentrations were determined using the BCA reagent (Vazyme). The fluorescence of samples was checked with a plate reader (Tecan Infinite TMM200) at 455 nm emission and 365 nm excitation.

### DNA methylation inhibitor treatment and DNA methylation analyses

The fruits were treated with 5-Aza [44]. One milliliter of 36 mM 5-Aza (Sigma) was dissolved in water and injected into fruit pericarp and pulp. One milliliter of water was injected into the other side of the fruit as a negative control (mock). Four injections were performed, 2 days apart. Twelve pummelos were injected, with three biological duplications.

The DNA was extracted following an earlier report with modification [45]. The EZ DNA Methylation-Startup Kit (Zymo Research) was used for bisulfite PCR according to the manufacturer’s instructions. Converted DNA was used for PCR amplification with ZymoTaq PreMix. The PCR products were cloned into a p-TOPO vector. Single-clone sequence data analysis was performed using Kismeth (http://katahdin.mssm.edu/kismeth/revpage.pl) and the methylation level of each fragment was calculated. For RT–PCR, HaeIII was used to digest DNA according to the operation manual. The PCR conditions were as follows: 95°C for 5 minutes; 29 cycles of 95°C for 30 seconds, 53°C for 30 seconds, and 72°C for 60 seconds, and 72°C for 5 minutes. The primers used for bisulfite sequencing are listed in Supplementary Data Table S7.

### Statistical analysis

Significance was evaluated using Student’s *t*-tests in Excel. Data were expressed as mean ± standard deviation of at least three repetitions.

## Acknowledgements

This project was financially supported a National Natural Science Foundation of China grant to Q.X. (Nos. 31925034 and 31872052), and key project of Hubei provincial Natural Science Foundation (2021CFA017).

## Author contributions

Q.X. conceived and supervised the study. Z.L. designed and completed the experiments and wrote the article. Y.H. completed the assembly of the genome. S.M., F.W., Y.L., X.M., P.B.A., Y.X. L.W., H.Z., and M.J.R. assisted in the experiments.

## Data availability

HCA genome sequences, HCA and LCA RNA-seq sequences, and their genome sequencing data can be accessed in the NCBI database using the accession numbers PRJNA796621 and PRJNA318855. HB, WSY, NP, and PP pummelo transcriptome data can be accessed in the Citrus Pan-genome to Breeding Database (http://citrus.hzau.edu.cn/).

## Conflict of interest

The authors declare that there are no conflicts of interest associated with this work.

## Supplementary data

Supplementary data is available at *Horticulture Research * online.

## Supplementary Material

supp_data_uhac175
