# Supplementary material for: The high-quality genome of pummelo provides insights into the tissue-specific regulation of citric acid and anthocyanin during domestication
Source: Hortic Res. 2022 Aug 4;9:uhac175. doi: 10.1093/hr/uhac175 (PMC9552194; doi:10.1093/hr/uhac175)
Supplement: supp_data_uhac175 [file supp_data_uhac175.zip › Supporting Information.docx]

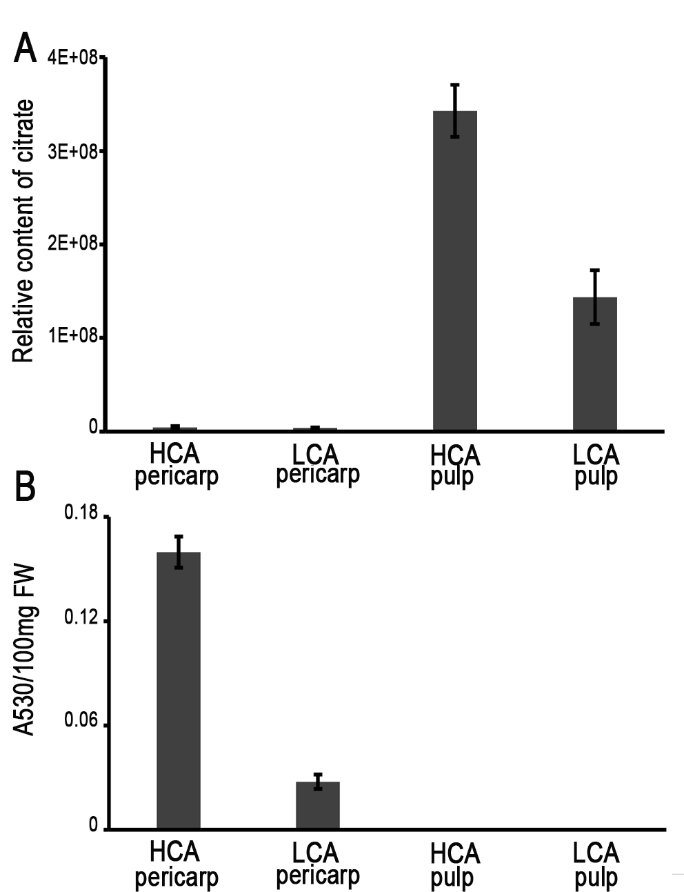


**Supplementary figure1.** Comparison of content of citric acid and anthocyanin in the pericarp and the pulp of HCA and LCA. A. The content of citric acid in pulp at DAF120 and pericarp at DAF10. B. The content of anthocyanin in the pulp at DAF120 and pericarp at DAF10


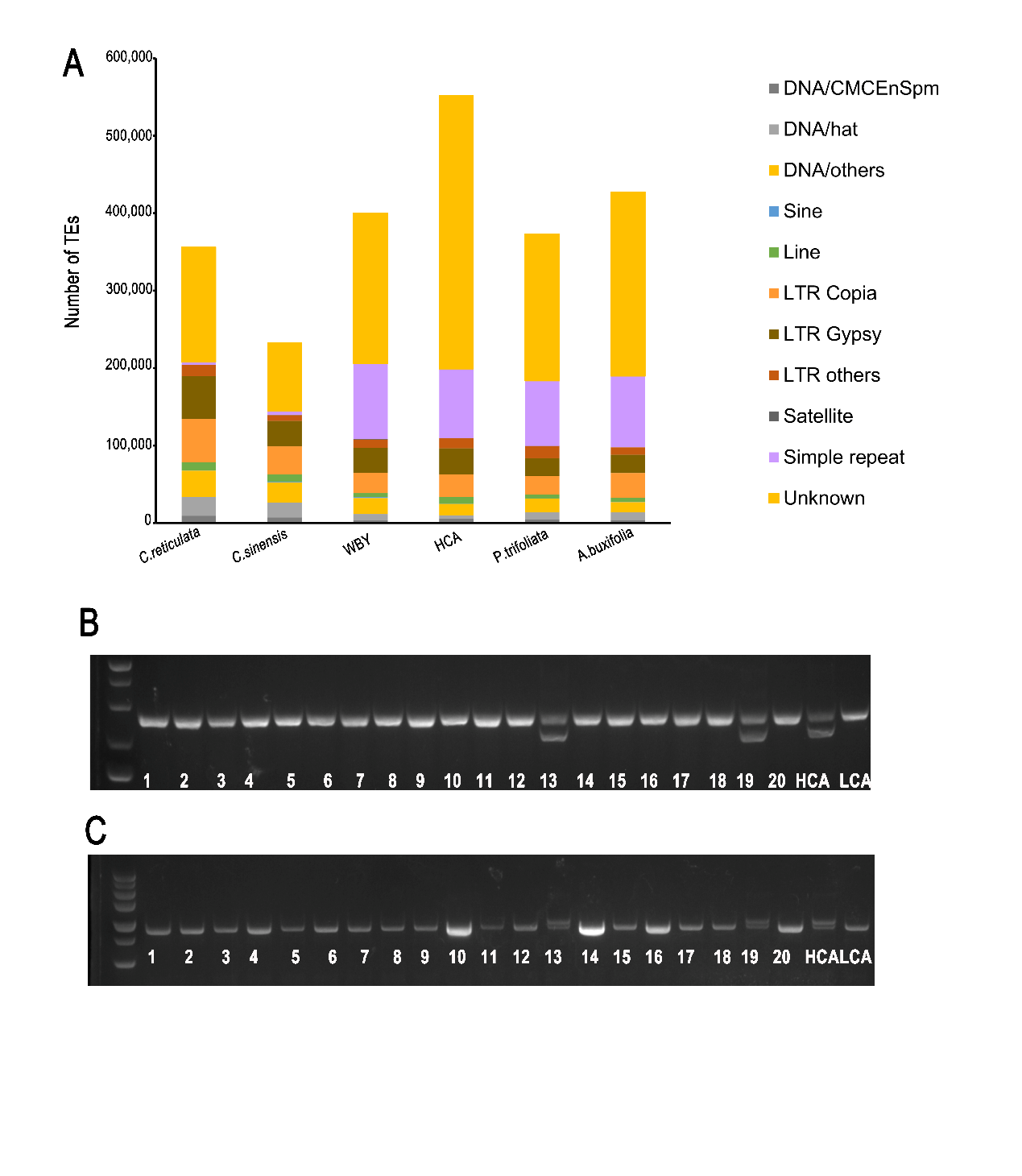


**Supplementary Figure2.** Comparison of the number of TEs among genomes of the six *Citrus* species and construction of InDel markers. A, The type and quantity of TEs were predicted among six *Citrus* species. B, C, The two InDel markers to distinguish HCA and LCA. 1-20 samples from the orchard were identified by using InDel markers. 13th and 19th samples are HCA others are LCA. The two rightmost columns are control.


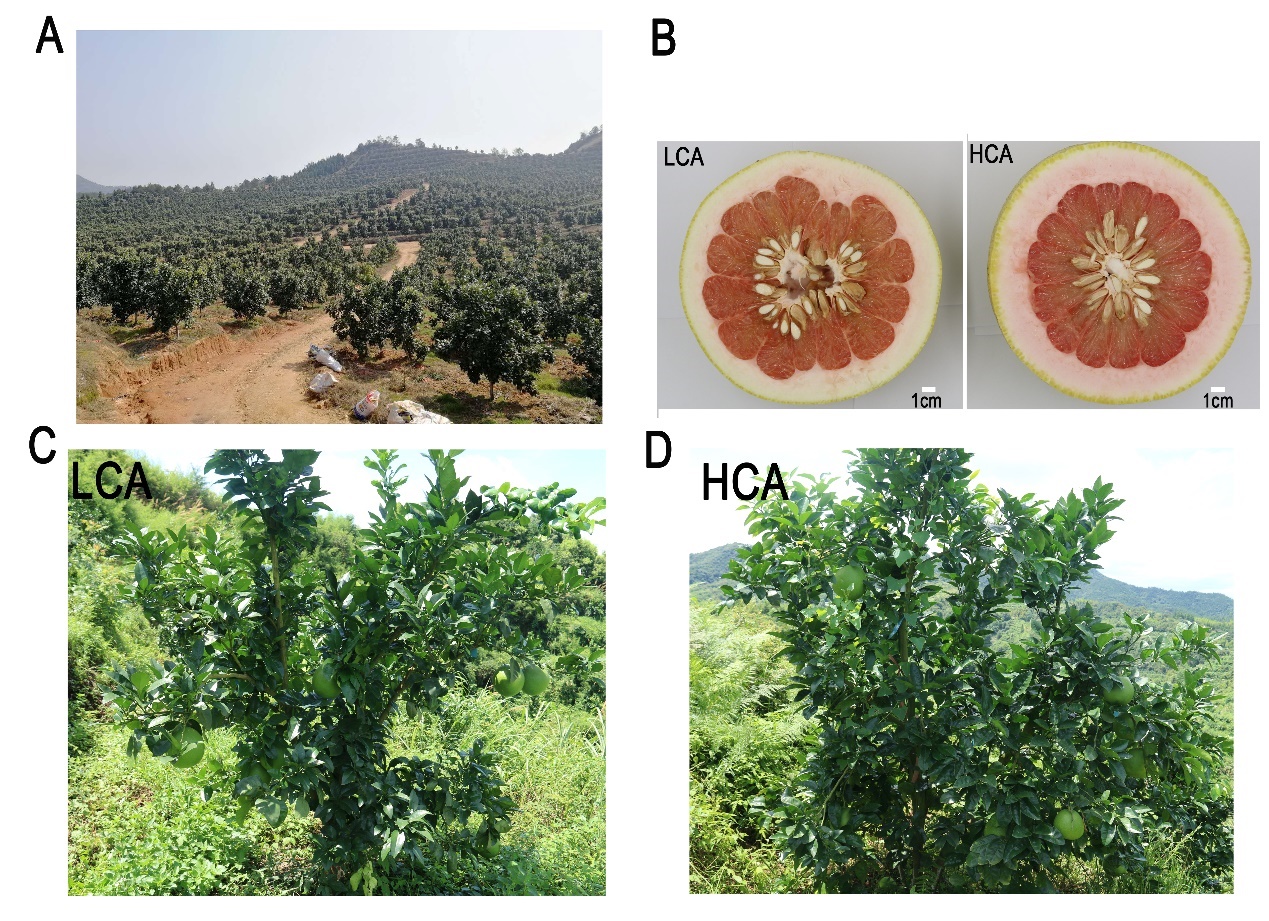


**Supplementary Figure 3.** Photos of two kinds of Majia pummelos. A, The aerial view of the commercial cultivation LCA model. B, Comparison of pulp in LCA and HCA. C, D, Comparison of status of growth between LCA and HCA.


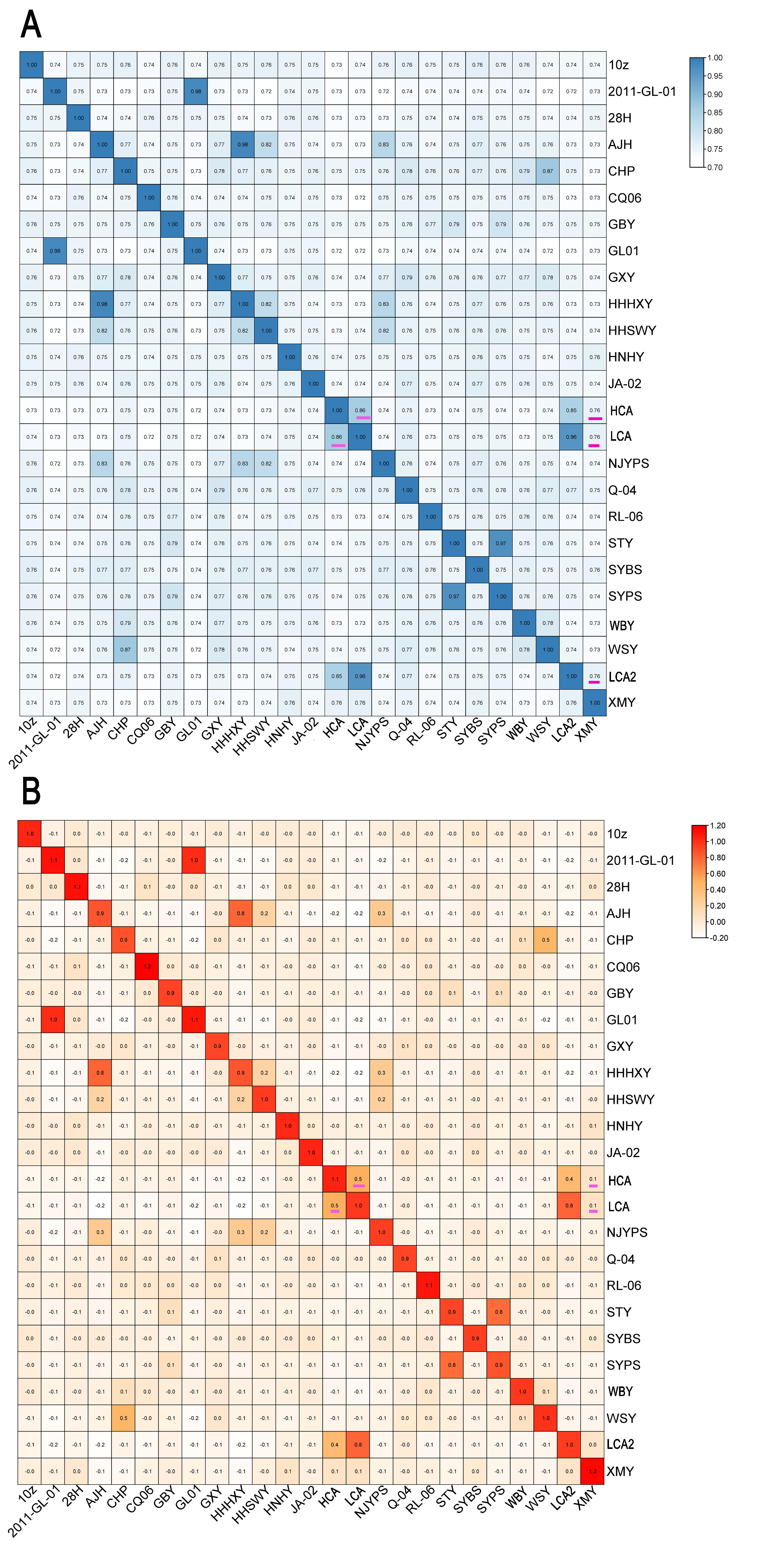


**Supplementary Figure4** Genetic relationship between LCA and HCA. B, IBS was calculated among 25 pummelo accessions based on SNP data. C, The value of the genetic relationship was calculated by gcta based on SNP. The accession IDs are corresponding to the information in Supplementary Table 7


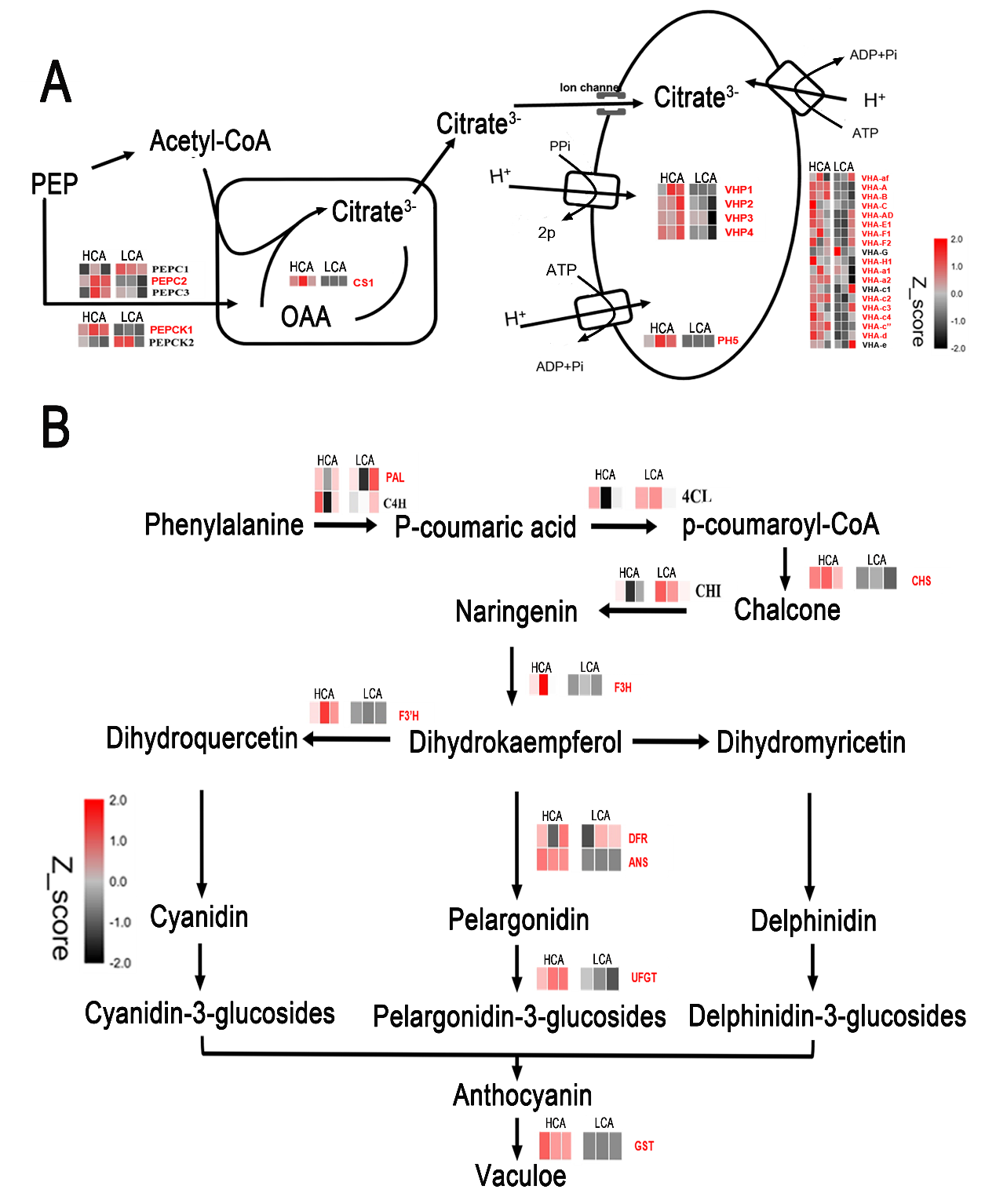


**Supplementary Figure5.** The expression level of pathway genes. A, The heatmap of the structural genes expression involved in citric acid accumulation. B, The heatmap of the structural genes expression involved in anthocyanin accumulation. The corresponding genes ID are provided in supplementary table 2.


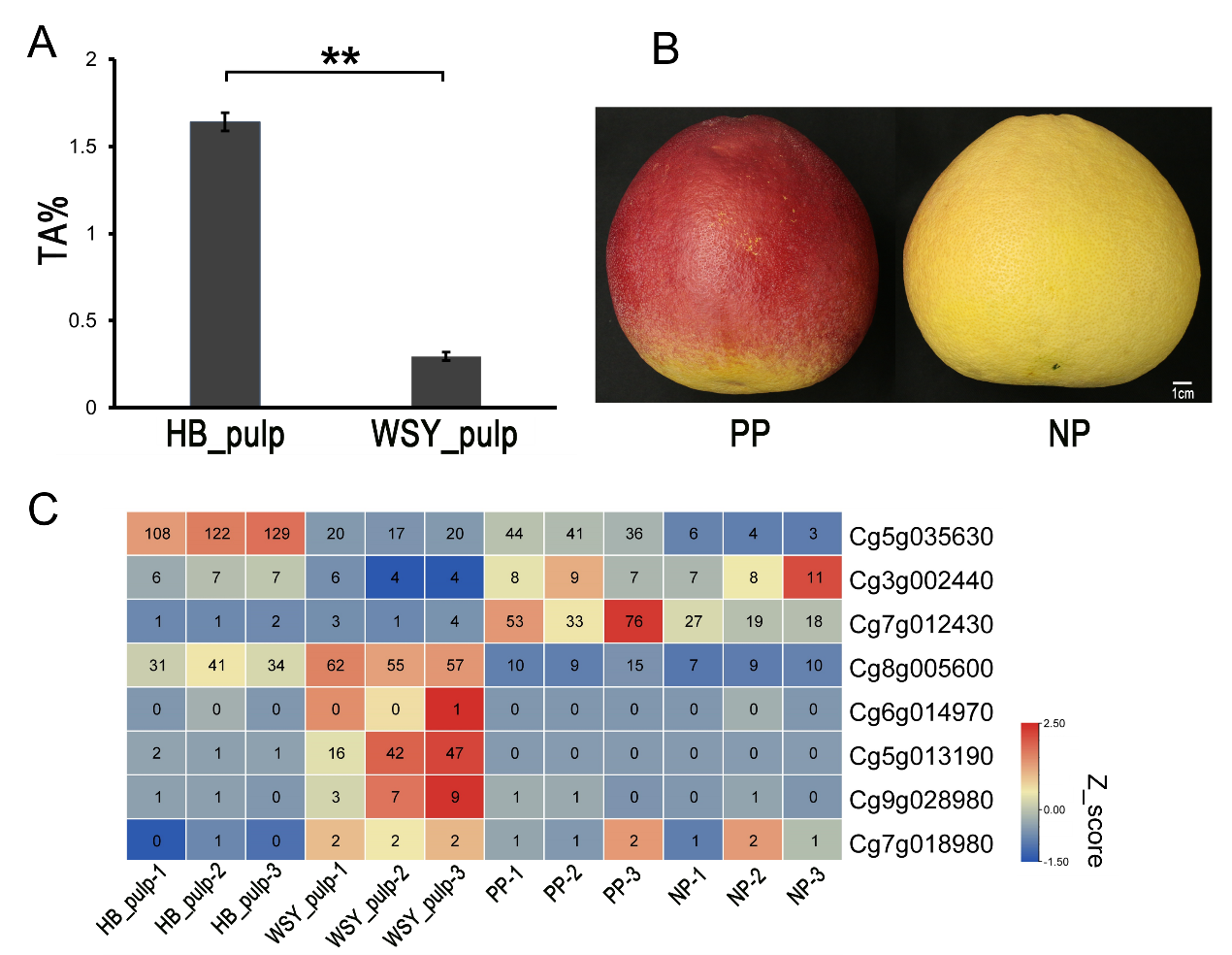


**Supplementary Figure6. The expression levels of eight candidate genes.** A, HB and WSY is a kind of high citric acid and acidless pummelo respectively. [Titratable](javascript:;) [acid](javascript:;) was measured at DAF120 in the pulp. Data are means ± SE (n = 3 biologically independent samples). ***P* < 0.01. B, Mature fruit of the purple pummelo (PP) and the normal pummelo (NP). These two kinds of pummelo had been reported in a previous study (Huang et al., 2018). C, The expression level of eight candidate genes in HB pulp at DAF120, WSY pulp at DAF120, PP pericarp at mature stage, and NP pericarp at mature stage are displayed. These data are FPKM and from Citrus Pan-genome to Breeding Database (<http://citrus.hzau.edu.cn/>).


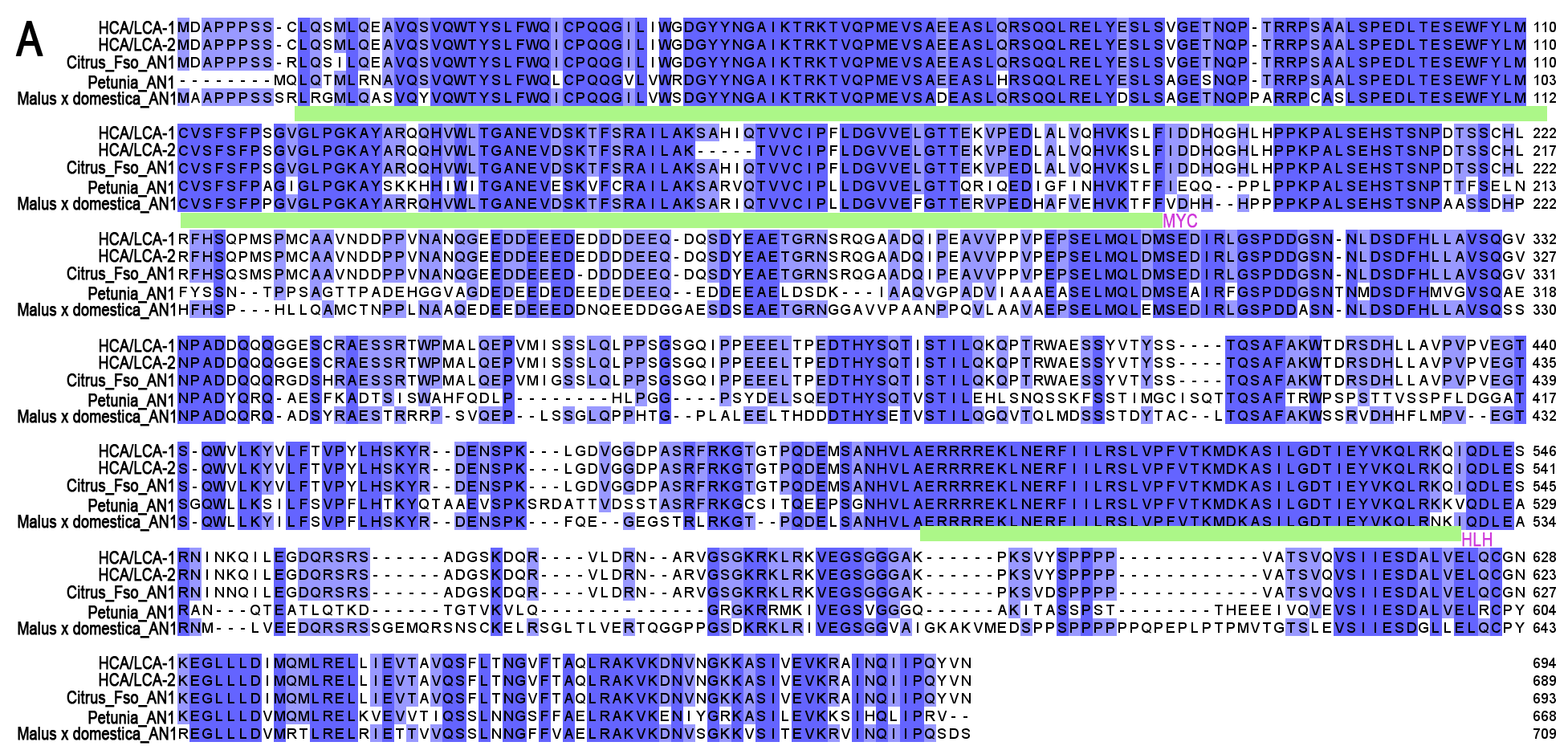


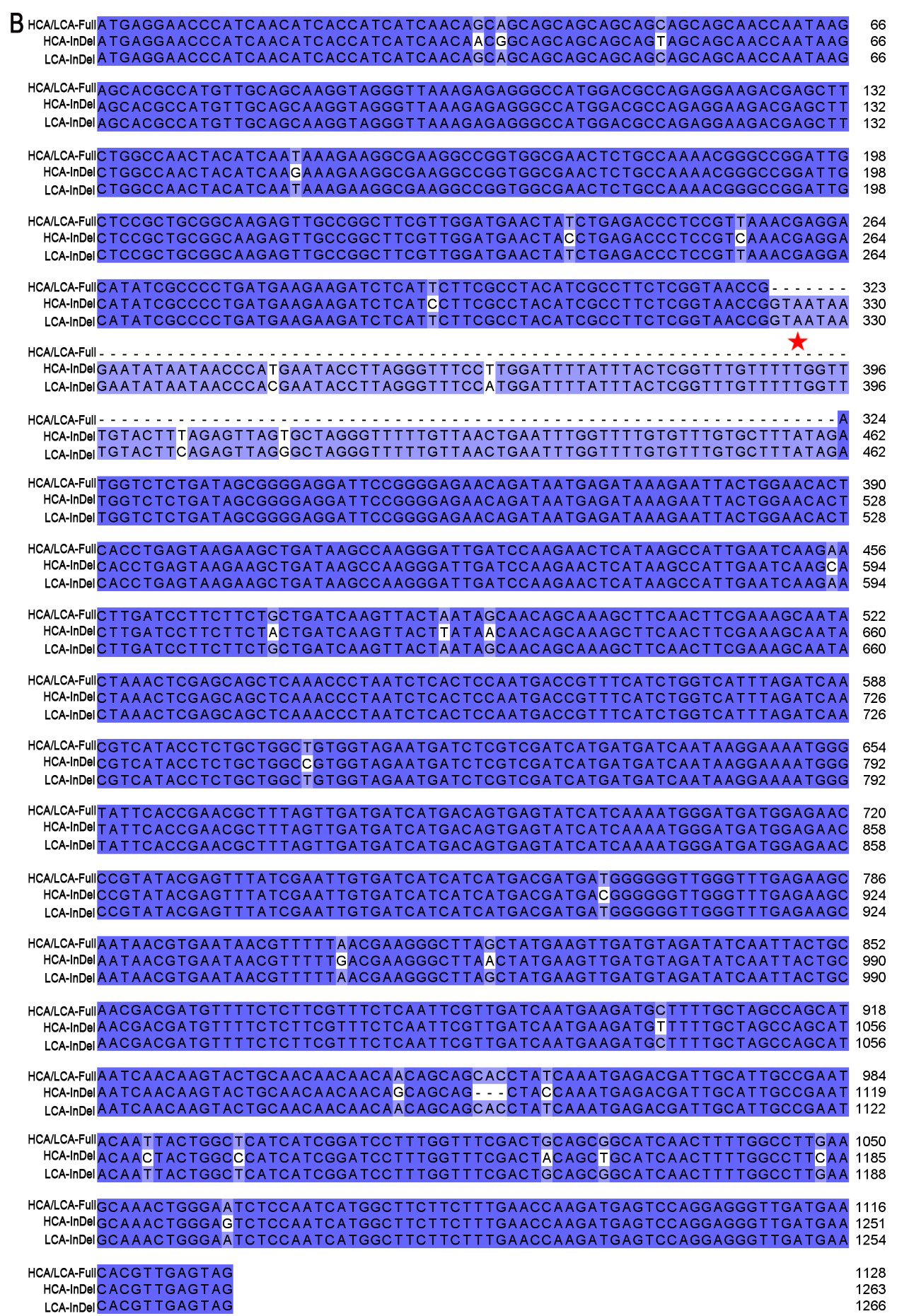


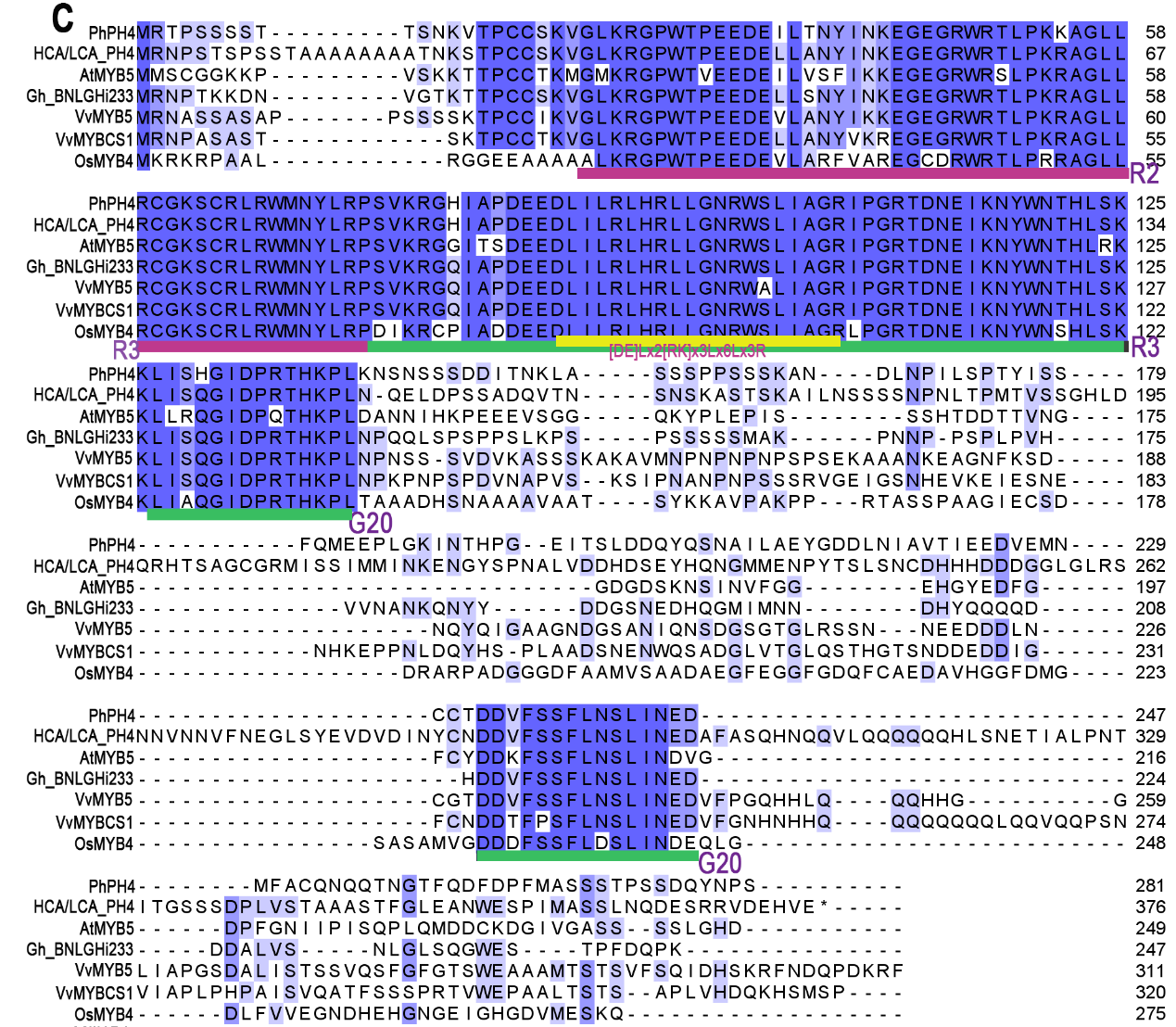


**Supplementary figure7.**  Analysing the sequence of *CgAN1*and *CgPH4*. A, Alignment of the sequence of *AN1* in HCA, LCA, Faris lemons, apple, and petunia. The green line implicated the conserved domain. B, The coding sequence of *CgPH4* in HCA and LCA. The red pentacle implicated the premature termination site. C, Comparing *CgPH4* coding sequence in HCA, LCA, and orthologous genes of *CgPH4* in Arabidopsis, Petunia, Vitis vinifera, and rice. G20: conserved sequence in G20 subgroup. The [DE]Lx2[RK]x3Lx6Lx3R is an interactional site between MYB and bHLH.


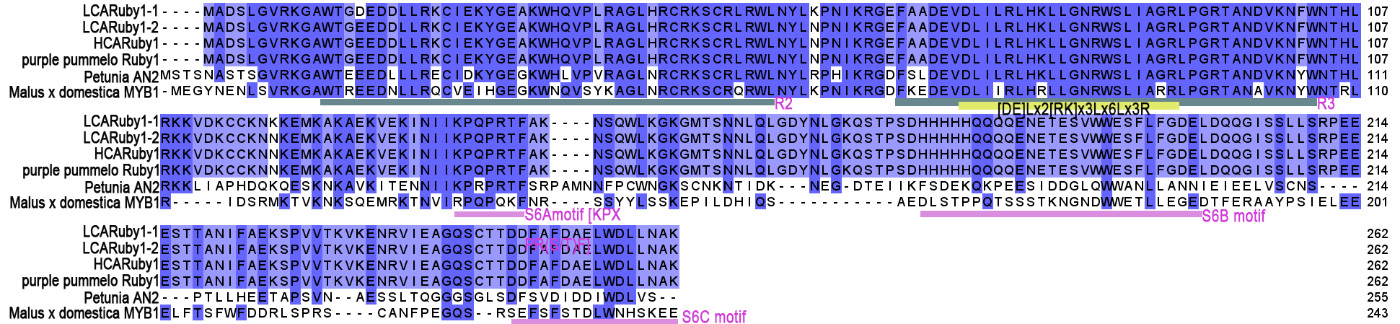


**Supplementary figure8.** Analysing the sequence of *Ruby1*. Alignment of the sequence of *Ruby1* in HCA, LCA, purple pummelo, apple, and petunia. The S6A also named KPXPR(S/T)F, S6B, S6C motif are correlated to anthocyanin accumulation.


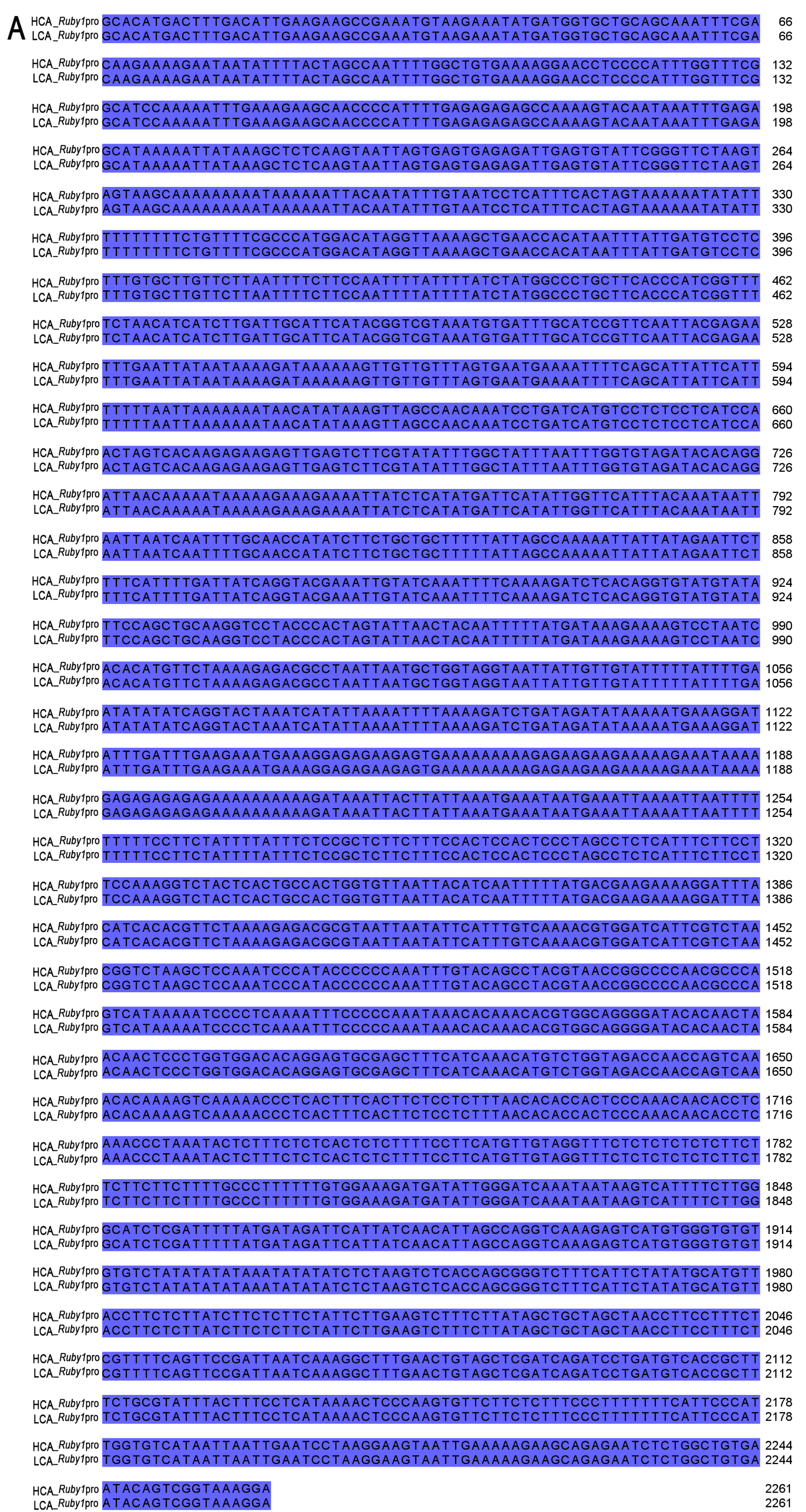


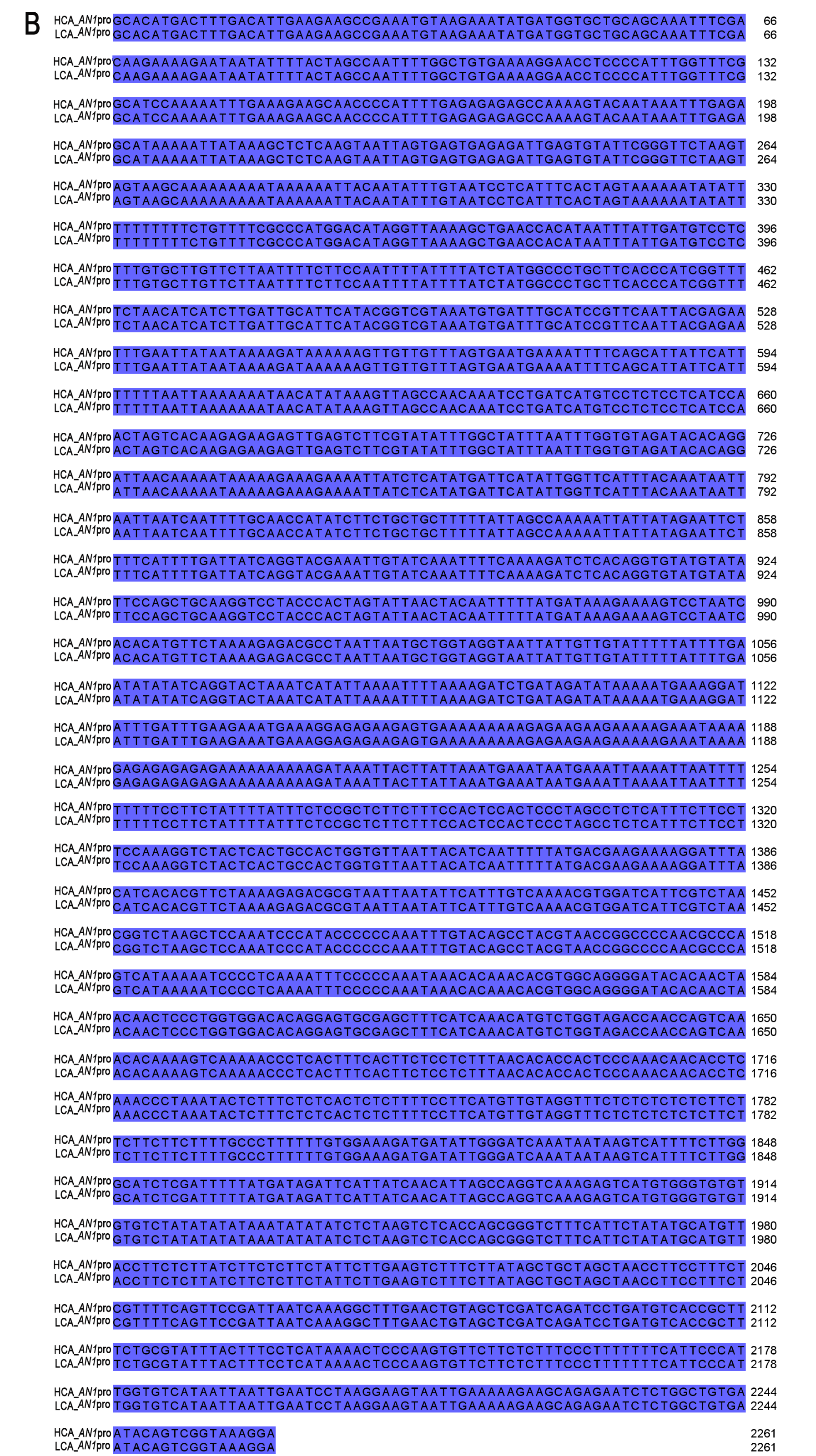


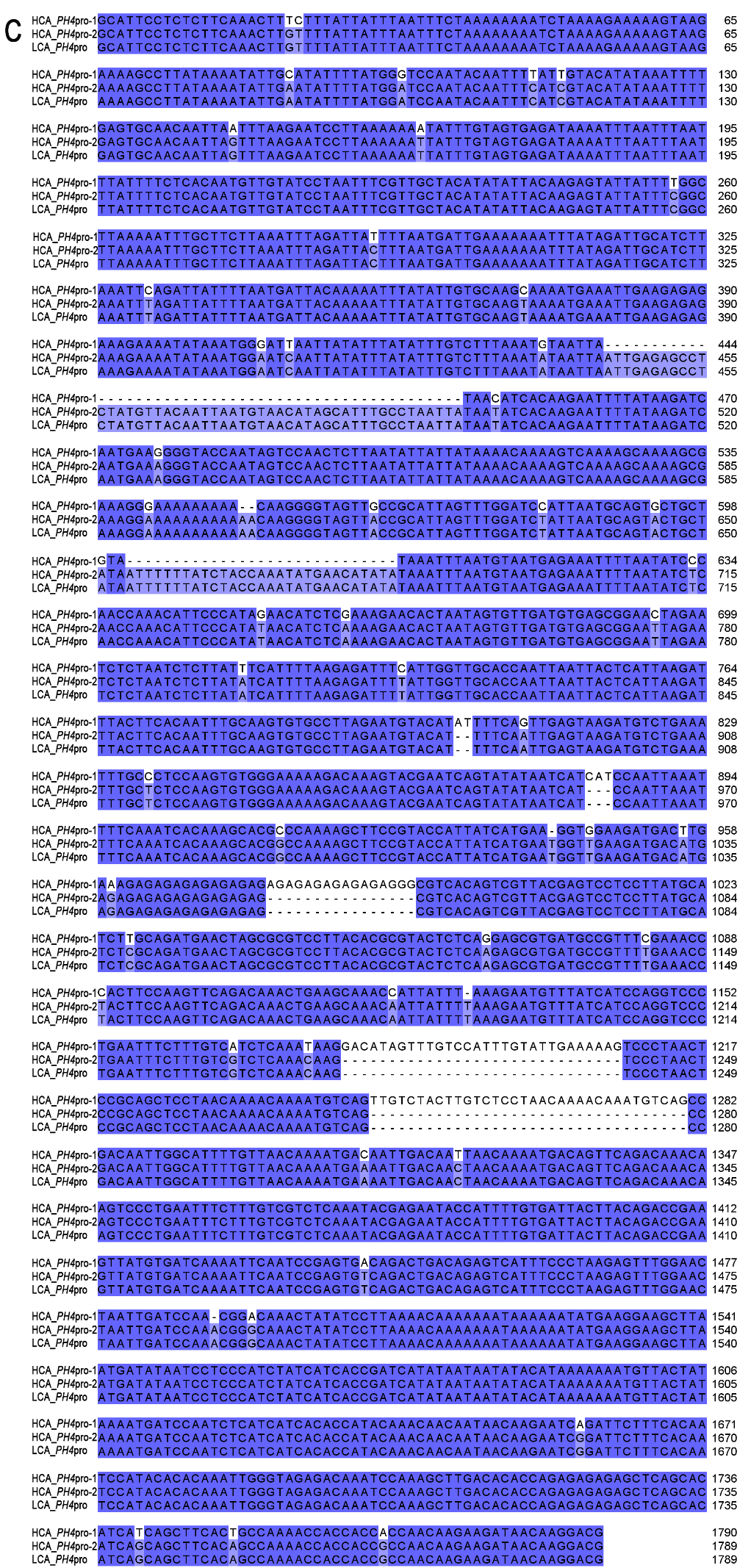


**Supplementary figure9.**  The sequence alignment of the promoter in *CgAN1*, *CgRuby1*, and *CgPH4*. A, Comparing the promoter of *CgRuby1* between HCA and LCA. B, Comparing the promoter of *CgAN1* between HCA. C, Comparing the promoter of *CgPH4* between HCA and LCA. The genotype of the *CgPH4* promoter in HCA is heterozygous.


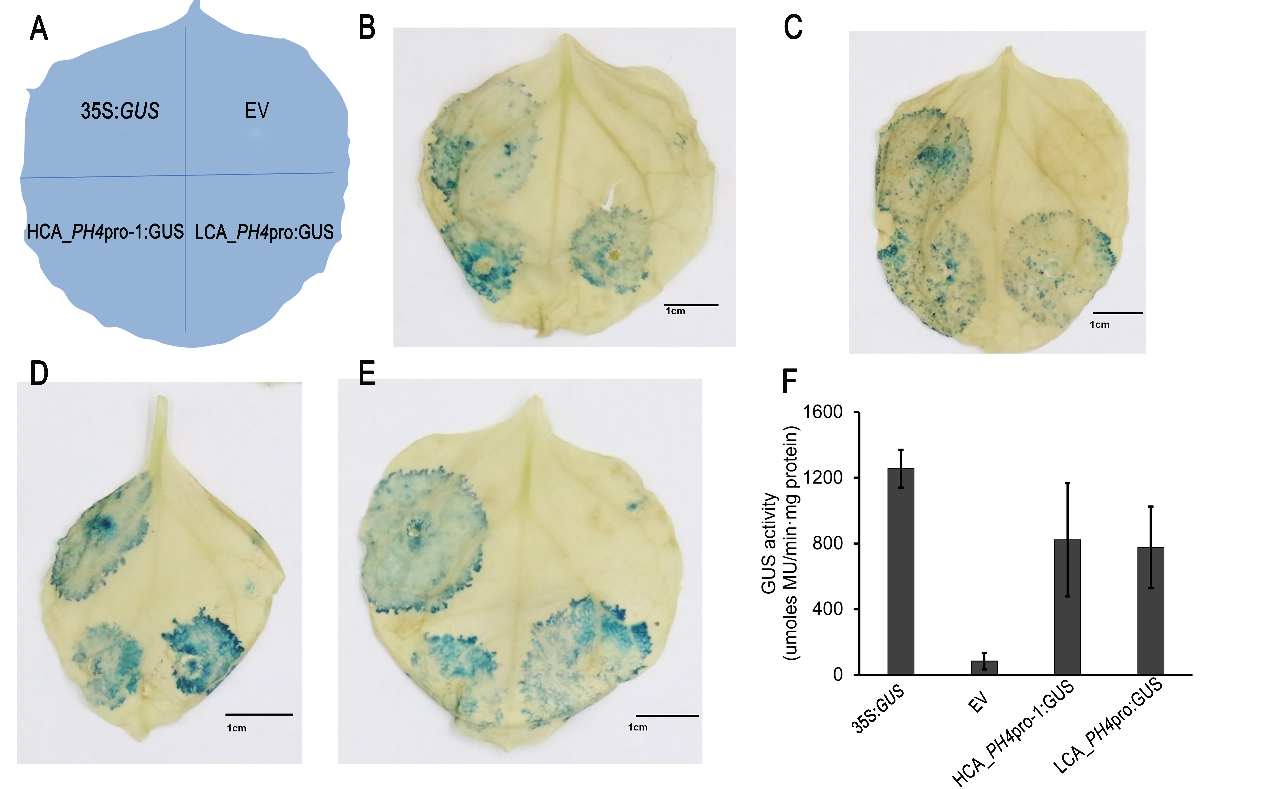


**Supplementary figure10.** The GUS assays for detecting the activity of the *CgPH4* promoter. A, A model shows, the injection position with different promoters driving GUS on tobacco leaves. B-E, The staining results of GUS protein by using x-gluc. F, The quantitative detection of GUS protein activity. The GUS reporter gene under the control of the CaMV35S promoter was used as the positive control (35S). The GUS reporter gene under the control of the non-promoter was used as the negative control (EV). Data are means ± s. e (n = 3 biologically independent replicates)


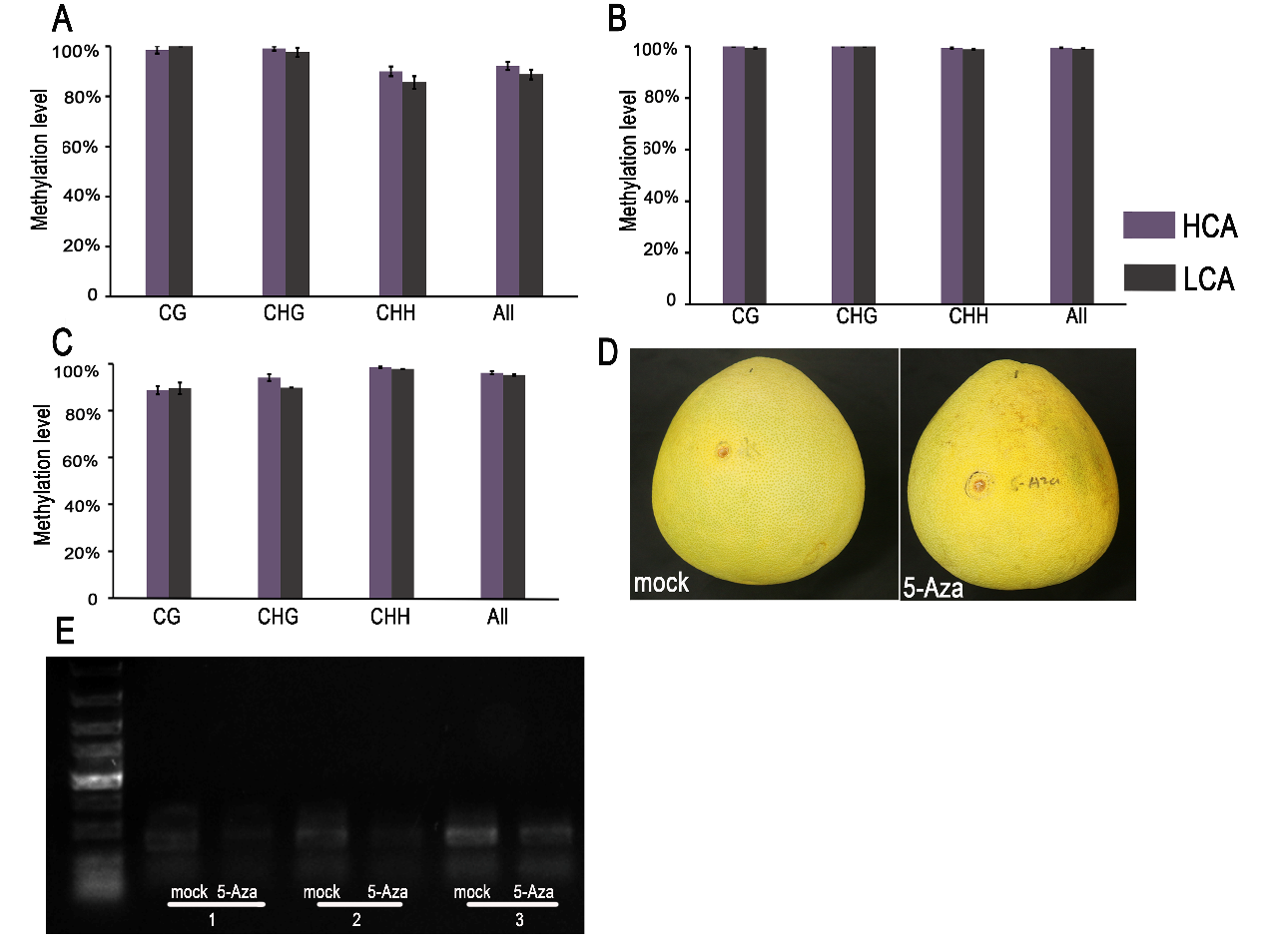


**Supplementary figure11.** Comparison of the cytosine methylation. A, B, C, Comparison of the cytosine methylation level on the -1 bp to -399 bp regions of *CgAN1* promoter in the pericarp*,* -1 bp to -771 bp regions of *CgPH4* promoter in the pulp and -1 bp to -510 bp regions of *CgRuby1* promoter in the pericarp of HCA and LCA. D, 5-Aza, and water are injected into both sides of the same LCA. The mock represents the water treatment. E, The promoter methylation of *CgAN1* was analysed using HaeIII after being treated by 5-Aza. The Y-axis represents the percentage methylation of each cytosine in the sequenced clones and four to fourteen clones were measured per sample.
